# Supplementary material for: An early diverging SQR enzyme in Antarctic Gloeobacterales indicates sulfide tolerance in thylakoid-lacking cyanobacteria
Source: Microbiol Spectr. 2026 May 13;14(6):e00423-26. doi: 10.1128/spectrum.00423-26 (PMC13228078; doi:10.1128/spectrum.00423-26)
Supplement: Supplemental materials — Supplemental methods, figures, and tables. [file spectrum.00423-26-s0001.docx]

**Materials and methods**

**Inference of SQR-I orthologous sequences**

We used OrthoFinder v2.5.5 (1) to identify the orthologous group corresponding to the SQR-I sequence. To work around OrthoFinder’s memory limit of 950 genomes, we first performed a dereplication of the 45,316 representative genomes of Terrabacteria species downloaded from GTDB r220 (2). The purpose of the dereplication was to select the most complete and least contaminated genomes while preserving the diversity of Terrabacteria in GTDB.

Genomes that passed the GUNC (3) contamination test were grouped by GTDB taxonomy order and ranked based on their CheckM2 (4) completeness minus CheckM2 contamination score. If an order contained only one genus, the two best genomes were selected; otherwise, one genome was chosen per order. This resulted in 1450 genomes without Cyanobacteria, which were added separately. The genome set for the Cyanobacteria phylum was sampled more extensively, resulting in 432 genomes. The list of cyanobacterial genomes was obtained from GTDB release r220 (2). One to two representative genomes were selected for each genus, with preference given to assemblies exhibiting high completeness (>80%) and low contamination (<10%) as assessed by CheckM2 (4). To ensure adequate representation of basal cyanobacterial lineages, all available genomes from NCBI corresponding to the orders *Gloeobacterales*, *Thermostichales*, *Pseudanabaenales*, and *Gloeomargaritales* were included. The *Pseudanabaenales* dataset was subsequently dereplicated using ToRQuEMaDA (v0.2.1) (5). The proteomes of the 1882 genomes were obtained from the NCBI database when available and otherwise predicted using Prodigal v2.6.3 (6). A ribosomal tree was subsequently inferred using the GEN-ERA toolbox (7), using IQ-TREE v2.4.0 (8) with the best model parameter (Q.YEAST+I+R10) and 1000 ultrafast bootstrap replicates. The ribosomal tree was then pruned using PARNAS v0.1.6 (9) by selecting 950 representative species, covering 90% of the overall diversity based on the generated diversity scores.

The orthogroup, computed with OrthoFinder v2.5.5 (1) on the 950 representative species, corresponding to SQR-I was identified based on sequence similarity with the *Synechocystis* sp. PCC 6803 substr. Kazusa Sll5036 (NCBI Protein Accession Number: BAD01806.1) using BLASTp searches (BLAST v2.9.0+ (10)). A phylogenetic tree of the SQR orthogroup was then generated using IQ-TREE v3.0.0 (8) with the best-fit model parameter (LG+I+G) and 1000 ultrafast bootstrap replicates. The sequences included in the phylogenetic tree were aligned using MAFFT v7.505 (11). The sites of the alignment were conserved if they had at most 30% gaps and the sequences with a minimum of 30% length were exported using ali2phylip.pl (D. Baurain;<https://metacpan.org/release/Bio-MUST-Core>). To avoid problems of paralogy during orthologous sequence inference, the sequences corresponding to SQR-I were located within the multigenic family tree based on their sequence similarity to the reference sequence Sll5036. A profile HMM (HMMER v3.4 (12)) was then generated from 11 cyanobacterial SQR-I orthologous sequences. This HMM profile was then used to search the proteomes of 432 Cyanobacteriota genomes to increase the sampling beyond the 950-sequence limit imposed by OrthoFinder’s memory constraints. This resulted in the discovery of three SQR-I sequences in *Gloeobacter* MAGs.

**SQR phylogenetic tree inference using GTDB representative species**

An HMM search was conducted across a dataset containing the conceptual proteomes of all representative species (107,237) from GTDB release r220 (2) and the proteomes of the three Gloeobacter MAGs. The proteomes of the selected genomes were inferred using Prodigal v2.6.3 (6). A preliminary tree was generated based on the sequences selected according to the criteria listed in **Supplementary Table 1** with Ompa-Pa v0.251830 (D. Baurain;<https://metacpan.org/dist/Bio-MUST-Apps-OmpaPa>) using FastTree v2.1.11 (14). The selection of the sequences used to build the tree was visualized with Ompa-Pa based on taxonomy, the fraction of the sequence covered by the alignment (alignment coverage) and number of copies per genome (**Supplementary Figure 1**). Selected sequences were annotated by performing BLASTp searches against a database comprising the different SQR types identified in a previous study (19). This tree (**Supplementary Figure 2**) contains a subtree represented exclusively by cyanobacterial SQR-I sequences. A phylogenetic tree of the proteins contained in this subtree was subsequently performed using IQ-TREE (8) with best-fit model (Q.PFAM+I+R8) and 1000 ultrafast bootstrap replicates (**Figure 1**). For each tree, sequences were aligned using MAFFT v7.505 (11). The sites of the alignment were conserved if they had at most 30% gaps and the sequences with a minimum of 30% length were exported using ali2phylip.pl (D. Baurain;<https://metacpan.org/release/Bio-MUST-Core>). The sequence similarity between the reference SQR-I (Sll5036) and the selected proteins was assessed with a BLASTp search (10). The associated supplementary data, including the sequence alignment and phylogenetic tree files, are available on Figshare (10.6084/m9.figshare.31333204).

We further performed an analysis on a reduced dataset (252 sequences, comprising 193 Cyanobacterial and 59 closely related non-Cyanobacterial sequences identified in the preliminary tree from Supplementary Figure 2). Maximum-likelihood analyses were conducted with IQ-TREE (8) under the C20 and LG+C20 models, whereas Bayesian inference was performed with PhyloBayes (20) under the CAT-Poisson + G4 model. All the inferred trees showed a basal position of the Gloeobacterales sequences in unrooted versions of the trees (Figshare: 10.6084/m9.figshare.31333204).

**An overview of the workflow is presented in Supplemental Figure 5**.

**Contamination assessment of the three *Gloeobacter* MAGs**

The three Gloeobacterales MAGs were subjected to contamination assessment using GUNC (3) and CheckM2 (4) within the GEN-ERA toolbox, and the results are summarized in **Supplemental Tables 3 and 4**.

**Functional predictions of *Gloeobacterales* SQR-I**

Functional domains were predicted using the web-based version of InterProScan 5 v106.0 (15). The multiple sequence alignment in **Supplemental Figure 4** was annotated based on (16).

**Logan kmer search**

Logan SRA search was performed using the first 1,000 bp of both Candidatus *Sivonenia alaskensis* GCA_949127895.1 and GCA_949127685.1 as queries in the web interface of Logan Search (https://logan-search.org/; (17)). The search was performed against the “All” database using the default similarity threshold (0.5).

**PsbA (D1) phylogenetic tree inference using Cyanobacteria species**

The tree was constructed following a similar approach as SQR-I. The orthogroup was identified based on sequence similarity with the two distinct copies of *Synechocystis* sp. PCC 6803 substr. Kazusa PsbA (slr1311 [NCBI Protein Accession Number: P16033.1], slr1181 [NCBI Protein Accession Number: P07826.3]) using BLASTp searches (BLAST v2.9.0+ (10)). A phylogenetic tree of the SQR orthogroup was then generated using IQ-TREE v3.0.0 (8) with the best-fit model parameter (LG+F+R5) and 1000 ultrafast bootstrap replicates. The sequences included in the phylogenetic tree were aligned using MAFFT v7.505 (11). The sites of the alignment were conserved if they had at most 30% gaps and the sequences with a minimum of 30% length were exported using ali2phylip.pl (D. Baurain;<https://metacpan.org/release/Bio-MUST-Core>). To avoid problems of paralogy during orthologous sequence inference, the sequences corresponding to PsbA were located within the multigenic family tree based on their sequence similarity to the two reference sequences. A profile HMM (HMMER v3.4 (12)) was then generated from 79 PsbA orthologous sequences. This HMM profile was then used to search the proteomes of 432 Cyanobacteriota genomes to increase the sampling beyond the 950-sequence limit imposed by OrthoFinder’s memory constraints. This resulted in the expansion of the initial dataset from 79 to 726 sequences. D1 variants were annotated based on their highest similarity with consensus sequences retrieved from Sheridan et al., 2020. Only the top BLASTp hit was retained, using an e-value threshold of 1 × 10⁻³. The associated supplementary data, including the sequence alignment and phylogenetic tree files, are available on Figshare (10.6084/m9.figshare.31333204).

**References**

1. Emms DM, Kelly S. 2019. OrthoFinder: phylogenetic orthology inference for comparative genomics. Genome Biol 20:238.

2. Parks DH, Chuvochina M, Rinke C, Mussig AJ, Chaumeil P-A, Hugenholtz P. 2021. GTDB: an ongoing census of bacterial and archaeal diversity through a phylogenetically consistent, rank normalized and complete genome-based taxonomy. gkab776. Nucleic Acids Research <https://doi.org/10.1093/nar/gkab776>.

3. Orakov A, Fullam A, Coelho LP, Khedkar S, Szklarczyk D, Mende DR, Schmidt TSB, Bork P. 2021. GUNC: detection of chimerism and contamination in prokaryotic genomes. 1. Genome Biology 22:178

4. Chklovski A, Parks DH, Woodcroft BJ, Tyson GW. 2023. CheckM2: a rapid, scalable and accurate tool for assessing microbial genome quality using machine learning. Nat Methods 20:1203–1212.

5. Léonard RR, Leleu M, Vlierberghe MV, Cornet L, Kerff F, Baurain D. 2021. ToRQuEMaDA: tool for retrieving queried Eubacteria, metadata and dereplicating assemblies. PeerJ 9:e11348.

6. Hyatt D, Chen G-L, LoCascio PF, Land ML, Larimer FW, Hauser LJ. 2010. Prodigal: prokaryotic gene recognition and translation initiation site identification. 1. BMC Bioinformatics 11:119.

7. Cornet L, Durieu B, Baert F, D’hooge E, Colignon D, Meunier L, Lupo V, Cleenwerck I, Daniel H-M, Rigouts L, Sirjacobs D, Declerck S, Vandamme P, Wilmotte A, Baurain D, Becker P. 2023. The GEN-ERA toolbox: unified and reproducible workflows for research in microbial genomics. GigaScience 12:giad022.

8.. Wong TKF, Ly-Trong N, Ren H, Baños H, Roger AJ, Susko E, Bielow C, Maio ND, Goldman N, Hahn MW, Huttley G, Lanfear R, Minh BQ. 2025. IQ-TREE 3: Phylogenomic Inference Software using Complex Evolutionary Models.

9. Markin A, Wagle S, Grover S, Vincent Baker AL, Eulenstein O, Anderson TK. 2023. PARNAS: Objectively Selecting the Most Representative Taxa on a Phylogeny. Syst Biol 72:1052–1063.

10. Camacho C, Coulouris G, Avagyan V, Ma N, Papadopoulos J, Bealer K, Madden TL. 2009. BLAST+: architecture and applications. 1. BMC Bioinformatics 10:421.

11. Katoh K, Standley DM. 2013. MAFFT Multiple Sequence Alignment Software Version 7: Improvements in Performance and Usability. 4. Mol Biol Evol 30:772–780.

12. Finn RD, Clements J, Eddy SR. 2011. HMMER web server: interactive sequence similarity searching. Nucleic Acids Res 39:W29–W37.

13. Lefort V, Desper R, Gascuel O. 2015. FastME 2.0: A Comprehensive, Accurate, and Fast Distance-Based Phylogeny Inference Program. Mol Biol Evol 32:2798–2800.

14. Price MN, Dehal PS, Arkin AP. 2010. FastTree 2 – Approximately Maximum-Likelihood Trees for Large Alignments. PLOS ONE 5:e9490.

15. Jones P, Binns D, Chang H-Y, Fraser M, Li W, McAnulla C, McWilliam H, Maslen J, Mitchell A, Nuka G, Pesseat S, Quinn AF, Sangrador-Vegas A, Scheremetjew M, Yong S-Y, Lopez R, Hunter S. 2014. InterProScan 5: genome-scale protein function classification. Bioinformatics 30:1236–1240.

16. Brito, J. A. et al. Structural and Functional Insights into Sulfide:Quinone Oxidoreductase,. Biochemistry **48**, 5613–5622 (2009).

17. Chikhi R, Raffestin B, Korobeynikov A, Edgar R, Babaian A. 2024. Logan: Planetary-Scale Genome Assembly Surveys Life’s Diversity. bioRxiv <https://doi.org/10.1101/2024.07.30.605881>.

18. Gascuel, O. BIONJ: an improved version of the NJ algorithm based on a simple model of sequence data. *Molecular Biology and Evolution* **14**, 685–695 (1997).

19. Marcia, M., Ermler, U., Peng, G. & Michel, H. A new structure‐based classification of sulfide:quinone oxidoreductases. *Proteins* **78**, 1073–1083 (2010).

20. Lartillot, N., Lepage, T., & Blanquart, S. (2009). PhyloBayes 3: A Bayesian software package for phylogenetic reconstruction and molecular dating. *Bioinformatics*, *25*(17), 2286–2288. <https://doi.org/10.1093/bioinformatics/btp368>

**Supplemental Figures and Tables**


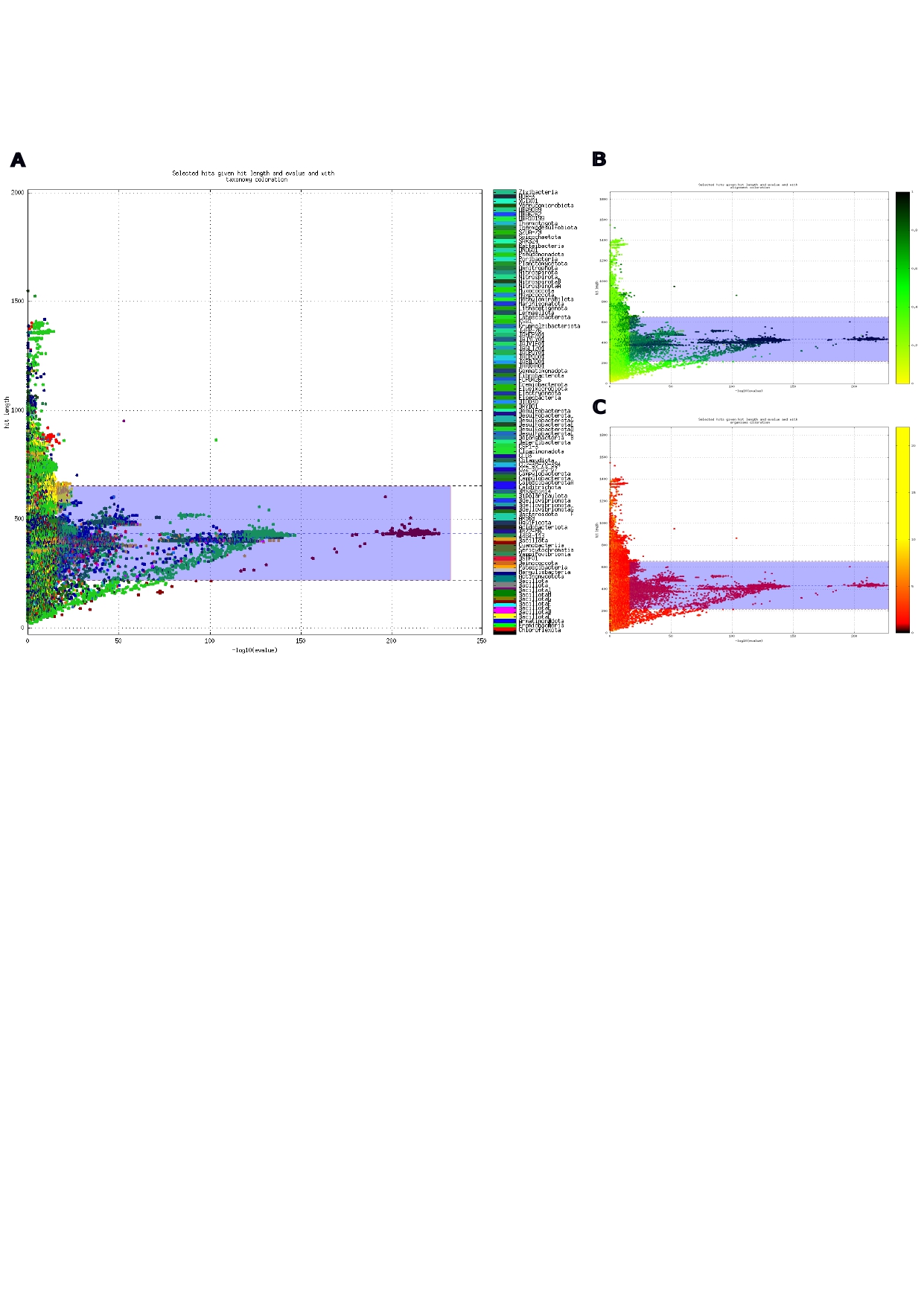


**Supplemental Figure 1: Visualization of the selection of the sequences similar to *Gloeobacterales* SQR-I with Ompa-Pa.** Matching sequences were plotted based on their sequence length in function of their -log10(e-value). The blue box corresponds to the selection. Sequences were visualized based on their (A) taxonomy, (B) alignment coverage and (C) number of copies per genome.

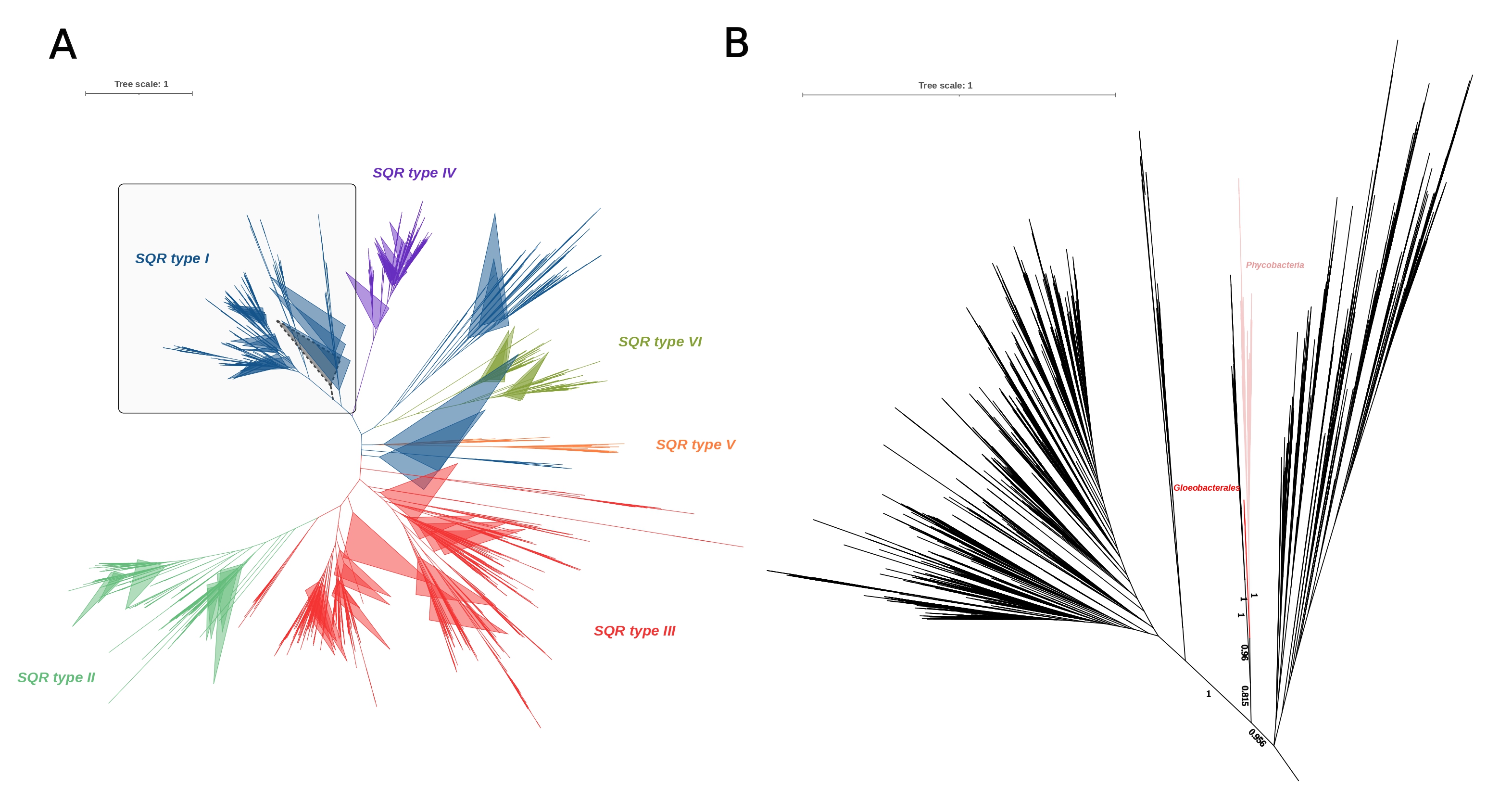
**Supplemental Figure 2: SQR phylogenetic tree prior to selection.**  (A) Preliminary multigenic family tree. The subtree corresponding to SQR-I sequences is shown in black. The tree was constructed from 19,081 sequences, corresponding to 453 unambiguously aligned positions, using FastTree with default parameters. The subtree highlighted in black represents the selected SQR-I subtree, corresponding to 192 sequences. (B) Subtree limited to cyanobacterial SQR-I sequences (highlighted in grey on panel A)). Gloeobacterales sequences are highlighted in red while the Phycobacteria sequences are highlighted in pink. The node support values are based on the Shimodaira-Hasegawa (SH) test (18).


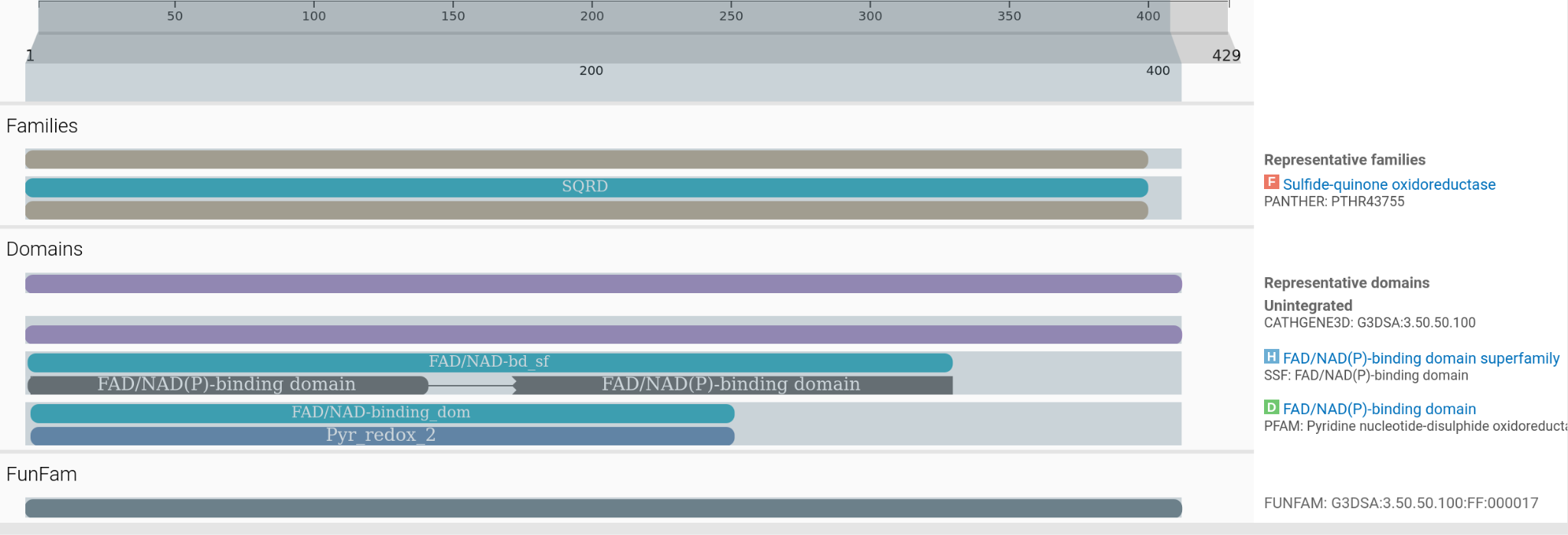


**Supplemental Figure 3: Functional domains of the SQR-I sequence from GCA_949127895.1.**


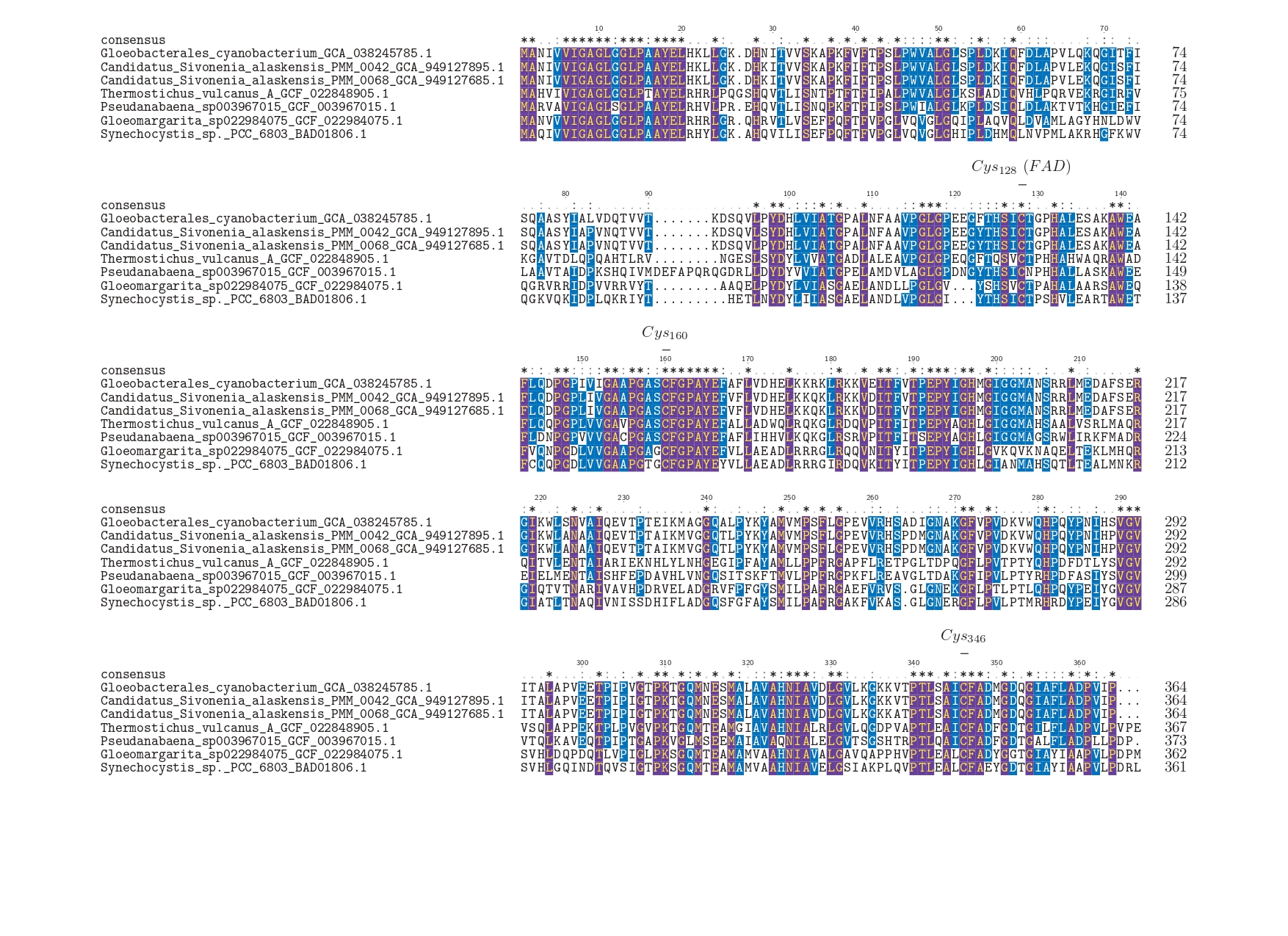


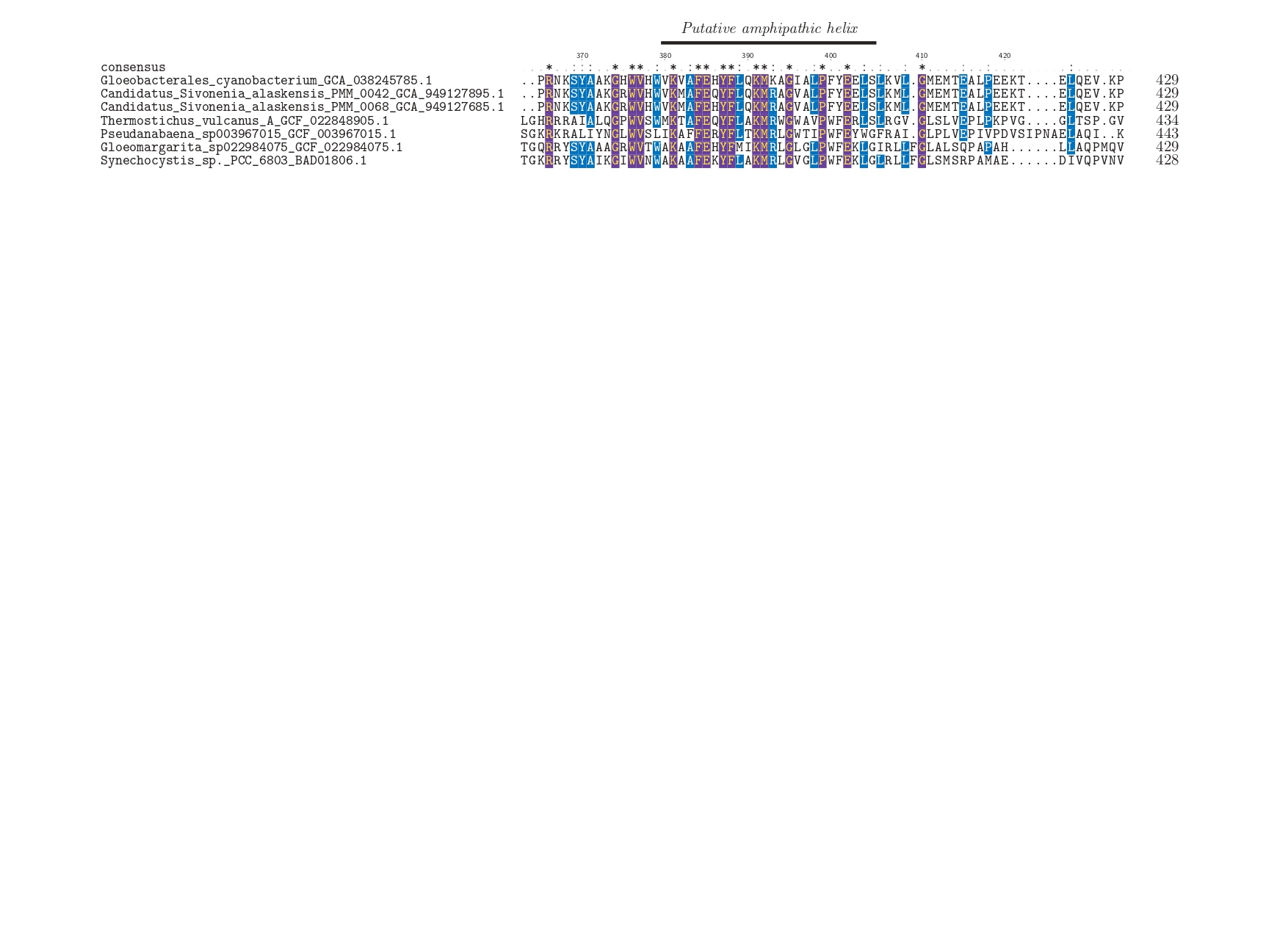


**Supplemental Figure 4: Amino acid sequence alignment of the three *Gloeobacter* SQR-I with SQR-I sequences of other basal lineages and the sequence of *Synechocystis* PCC 6803 substr. Kazusa Sll5036.** The residues playing a direct role in the catalytic mechanism of the enzyme, by covalently binding FAD or creating a sulfide bond, are annotated. The C-ter putative amphipathic helix supposedly anchors the protein within a membrane (16). Residues that are 100% conserved are indicated by an asterisk (*); residues conserved in more than 50% of the sequences are indicated by a colon (:); and poorly conserved residues are indicated by a dot (·).


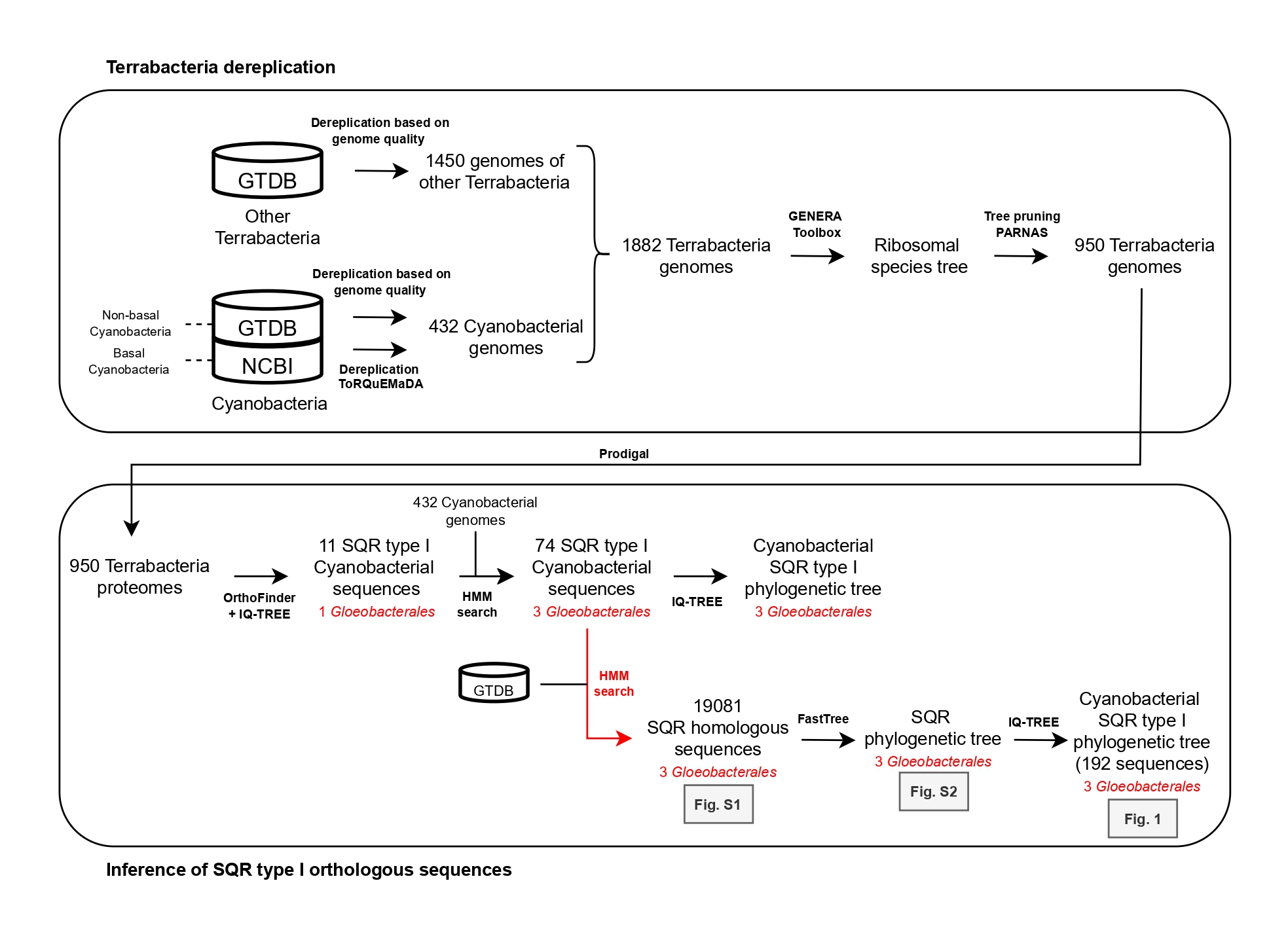
**Suppl​​emental Figure 5: Workflow overview.**

| **Accession** | **Biosample** | **Longitude** | **Latitude** | **Country** | **Cold criterion** |
| --- | --- | --- | --- | --- | --- |
| SRR7514503 | SAMN09633317 | -111.338851 | 58.778056 | Alberta, Canada | Subarctic (50°–66°N) |
| ERR10836081 | SAMEA112464045 | 158.8320278 | -54.71655556 | Macquarie Island, Australia | Subantarctic island (explicit polar region) |
| ERR10836083 | SAMEA112464047 | 37.58983333 | -46.94294444 | Marion Island, South-Africa | Subantarctic island (explicit polar region) |
| ERR12143029 | SAMEA114504931 | -73.41625 | 47.81176 | St. Maurice River, Canada | Temperate (<50°N, non-polar) |
| ERR12143032 | SAMEA114504934 | -73.41625 | 47.81176 | St. Maurice River, Canada | Temperate (<50°N, non-polar) |
| ERR3772646 | SAMEA6430891 | 102.289 | 72.399 | Tundra, Russia | Polar (>66°N, Arctic tundra) |
| SRR10595505 | SAMN13483817 |  |  | Artic | Polar (explicit Arctic location) |
| SRR3480285 | SAMN04958484 | -170.25 | 57.18 | Lake Hill Alaska | Subarctic (50°–66°N) |
| SRR3985421 | SAMN05421611 | -156.61 | 71.2999 | Barrow, Alaska | Polar (>66°N) |
| SRR3985424 | SAMN05422056 | -156.61 | 71.2999 | Barrow, Alaska | Polar (>66°N) |
| SRR3985527 | SAMN05422055 | -156.61 | 71.2999 | Barrow, Alaska | Polar (>66°N) |

**Supplemental Table 1: Information on SRA entries identified by Logan search.**

| **Criteria** | **Cyanobacteria sampling** | **GTDB representative species** |
| --- | --- | --- |
| Maximum copy number | 3 | 10 |
| Maximum sequence coverage | 1 | 1 |
| Maximum –log(e-value) | 241 | 233 |
| Maximum sequence length | 515 | 646 |
| Minimum copy number | 1 | 1 |
| Minimum sequence coverage | 0 | 0 |
| Minimum –log(e-value) | 154 | 18 |
| Minimum sequence length | 282 | 215 |

**Supplemental Table 2: Criteria applied for the selection of similar SQR-I sequences using Ompa-Pa.**

| **genome** | **n_genes_called** | **n_genes_mapped** | **n_contigs** | **taxonomic_level** | **proportion_genes_retained_in_major_clades** | **genes_retained_index** | **clade_separation_score** | **contamination_portion** | **n_effective_surplus_clades** | **mean_hit_identity** | **reference_representation_score** | **pass.GUNC** |
| --- | --- | --- | --- | --- | --- | --- | --- | --- | --- | --- | --- | --- |
| GCA_038245785.1 | 5142 | 4612 | 224 | phylum | 0.92 | 0.82 | 0.26 | 0.12 | 0.26 | 0.62 | 0.51 | TRUE |
| GCA_949127685.1 | 2942 | 2658 | 344 | phylum | 0.94 | 0.85 | 0.16 | 0.06 | 0.12 | 0.62 | 0.53 | TRUE |
| GCA_949127895.1 | 3042 | 2742 | 380 | phylum | 0.94 | 0.84 | 0.34 | 0.07 | 0.15 | 0.62 | 0.52 | TRUE |

**Supplemental Table 3: GUNC metrics for contamination assessment of the three *Gloeobacter* MAGs.**

| **Name** | **Completeness** | **Contamination** | **Completeness_Model_Used** | **Translation_Table_Used** |
| --- | --- | --- | --- | --- |
| GCA_038245785.1 | 98.24 | 1.42 | Gradient Boost (General Model) | 11 |
| GCA_949127685.1 | 94.6 | 0.45 | Gradient Boost (General Model) | 11 |
| GCA_949127895.1 | 92.74 | 1.93 | Gradient Boost (General Model) | 11 |

**Supplemental Table 4: CheckM2 metrics for contamination assessment for the three *Gloeobacter* MAGs.**
